# Current Nucleic Acid Extraction Methods and Their Implications to Point-of-Care Diagnostics

**DOI:** 10.1155/2017/9306564

**Published:** 2017-07-12

**Authors:** Nasir Ali, Rita de Cássia Pontello Rampazzo, Alexandre Dias Tavares Costa, Marco Aurelio Krieger

**Affiliations:** ^1^Departamento de Engenharia de Bioprocessos e Biotecnologia, Universidade Federal do Paraná (UFPR), Curitiba, PR, Brazil; ^2^Instituto de Biologia Molecular do Paraná (IBMP), Fiocruz, Curitiba, PR, Brazil; ^3^Instituto Carlos Chagas (ICC), Fiocruz, Curitiba, PR, Brazil

## Abstract

Nucleic acid extraction (NAE) plays a vital role in molecular biology as the primary step for many downstream applications. Many modifications have been introduced to the original 1869 method. Modern processes are categorized into chemical or mechanical, each with peculiarities that influence their use, especially in point-of-care diagnostics (POC-Dx). POC-Dx is a new approach aiming to replace sophisticated analytical machinery with microanalytical systems, able to be used near the patient, at the* point of care* or* point of need*. Although notable efforts have been made, a simple and effective extraction method is still a major challenge for widespread use of POC-Dx. In this review, we dissected the working principle of each of the most common NAE methods, overviewing their advantages and disadvantages, as well their potential for integration in POC-Dx systems. At present, it seems difficult, if not impossible, to establish a procedure which can be universally applied to POC-Dx. We also discuss the effects of the NAE chemicals upon the main plastic polymers used to mass produce POC-Dx systems. We end our review discussing the limitations and challenges that should guide the quest for an efficient extraction method that can be integrated in a POC-Dx system.

## 1. Introduction

Nucleic acid extraction (NAE) is one of the most pivotal steps in molecular biology, being routinely used in many areas of the biological and medical sciences, as this procedure marks a starting point in any molecular diagnostic kit [1]. This crucial procedure has been known for over a century and has developed substantially over the last decades. However, some progress still has to be achieved so that NAE protocols leave the laboratory settings into the “real world” of point-of-care diagnostics (POC-Dx).

Nowadays, it is known that intracellular nucleic acids (NAs) may be broadly categorized as genomic (or chromosomal), plasmids, and different types of RNAs [2]. Although RNAs possess uracil while DNAs present thymine [3], nucleic acids exhibit similar basic biochemical properties but might have quite distinct tridimensional structures (genomic, plasmid, tRNA, mRNA, rRNA, etc). However, despite the structural differences, the most commonly used methods described in the present text can be applied to DNA in its many organizational formats (chromosomal, plasmid, etc.), as well as RNA and its multidimensional formats (mRNA, rRNA, tRNA, miRNA, etc.) with minor modifications [1, 4, 5].

NAE can be roughly divided into four steps, which can be modulated depending on the sample and downstream applications: (i) cell disruption; (ii) removal of membrane lipids, proteins, and other nucleic acids, (iii) nucleic acid purification/binding from bulk; and (iv) nucleic acid concentration [6].

Cell disruption or disintegration can be achieved by physical and/or chemical methods, whose main aim is to disrupt the cell wall and/or cellular membranes. Disruption methods are mainly based on properties of the sample and for this purpose a wide range of tools and approaches are used either alone or combined to achieve tissue/cell disruption [7]. Lytic enzymes, chaotropic agents, and different types of detergents are the main components of chemical lysis, while mechanical method disrupts the cells by grinding, shearing, bead beating, and shocking [8]. It is interesting to note that if one technique does not yield good results, another might prove successful. Osmotic shock methods have yielded, in certain cases, better results than common NA purifications protocols such as phenol-chloroform extraction and bead beating [9]. Not only is cell disruption important for DNA extraction, but it also plays a crucial role in the biopharmaceutical industry, as many recombinant proteins and other important constituents of the cell can be recovered through this process [10–12]. Another approach for cell disruption is the use of different methods in combination. A good example is the case for enzymatic lysis, where many protocols use proteases to free the NA from its protective protein scaffold. Also, the inactivation of cellular nucleases that come free into solution in order to protect the new protein-free NA is crucial [13]. A combination of detergents and chaotropic salts in a single solution is used to solubilize cell wall and or cell membrane and inactivate intracellular nucleases [14, 15]. Mechanical disruption, on the other hand, makes use of force to extract out constituents of the cell. A classic example of grinding in biosciences is the use of mortar and pestle [6], which is nowadays optimized with the use of liquid nitrogen (when allowed by the sample). Cells walls can also be disrupted by the shock waves created by rapid changes in pressure elicited by sonication or cavitation [16–18]. Other mechanical tools available for cell disruption are shearing, which use a tangential force to make a hole in the cell [6], and bead beating, which uses different glass or steel beads to rupture tough cell wall as mentioned by Bunge et al. [19]. These processes are briefly summarized in Table 1, with consolidated examples.

NAE methods encompass extraction of both DNA and RNA but can be more broadly characterized into chemically driven or solid-phase methods; both contain the four steps mentioned above [1, 4, 5]. In the next sections, we will review the working principle of and/or rationale for the main methods used nowadays in the biological and medical sciences. Since molecular diagnostics rely heavily on techniques that start with NAE, we will also discuss some of the basic features of devices available for POC-Dx, culminating with the challenges and limitations of adapting NAE methods to point-of-care diagnostic tests.

## 2. Chemically Driven Methods

These methods rely on biochemical properties of the cellular components to elicit the desired molecular separation and might exhibit preference or exclusivity in extracting DNA or RNA, depending on its intrinsic characteristics.

### 2.1. Cesium Chloride (CsCl) Gradient Centrifugation with Ethidium Bromide (EtBr)

This technique is mainly based on the phenomenon of buoyant and specific density. Ethidium bromide (EtBr) is an intercalating agent, thus reporting the location of the double-stranded DNA under UV-light and allowing the easy visual separation of the supercoiled and nonsupercoiled DNA molecules. The basic mechanism by which EtBr separates the two molecules is decreasing the buoyant density of comparatively linear molecules [20]. After the ultracentrifugation, CsCl has to be dialyzed of the collected DNA. The method can be used to extract DNA from bacteria, although a large-scale culture is needed [21]; this method can be used for purification of various forms of DNA, such as chromosomal, plasmid DNA, rDNA, or mitochondrial DNA [22]. Being sensitive and provider of good yields of pure DNA, the method is laborious, time-consuming, and costly as compared to other purification protocols. Furthermore, EtBR can affect downstream applications, such as PCR, cloning, and DNA sequencing [23]. There is concern about using EtBr, which is known to cause genotoxicity and frame shift mutations. For mice, nontoxic doses up to 50 mg/kg have been used, for cattle, up to 1 mg/kg of body weight. However, the concentration used in gel staining solutions (0.25–1 *µ*g/mL) is below the level of toxicity, even though care is suggested in handling EtBr [23, 24].

### 2.2. Guanidinium Thiocyanate-Phenol-Chloroform Extraction

A guanidinium thiocyanate- (GuSCN-) phenol-chloroform mixture allows for RNA extraction in a single-step procedure, as demonstrated by Chomczynski and Sacchi [25]. Prior to the development of guanidinium method, phenol extraction was normally used for extraction in a two-step, laborious process. The method was modified successively over time, starting from Ullrich et al. [26] who used guanidinium thiocyanate instead of guanidinium chloride for RNA isolation, followed later on by Chirgwin et al. in 1979 [27] using GuSCN combined with extended hours of ultracentrifugation and a CsCl cushion. In order to enhance the quality of the final nucleic acid, the technique was improved by using guanidinium thiocyanate and phenol-chloroform with a shorter centrifugation time [28]. Despite being less soluble in water than guanidine hydrochloride, another common salt of guanidine, GuSCN has stronger denaturing properties because both its ions are chaotropic.

The basic principle of the method is the separation of RNA from DNA and proteins after extraction with an acidic solution, which consists mainly of GuSCN, sodium acetate, phenol, and chloroform, followed by centrifugation. Total RNA remains in the upper aqueous phase, while most of DNA and proteins part remain either in the interphase or in the lower organic phase under acidic condition. Total RNA is then recovered through precipitation by isopropanol and can be used for subsequent process. The original method was carried out in mammalian tissue but, later on, it has been used for plants with some modification [29], animals [27], and cultured cell tissues as well [28, 30]. Optimum pH plays a critical role in the separation process as DNA partitions to the organic phase under acidic condition (pH 4–6) or to the aqueous phase at neutral pH (pH 7-8). The main drawback of this method is that phenol and chloroform are both hazardous chemicals [28]. This reagent is commercially available with different names, such as Sigma-Aldrich TRI Reagent® and Thermo Fisher TRIzol® Reagent. High purity and yield of the extracted NA are the hallmark of this procedure.

### 2.3. Cetyltrimethylammonium Bromide (CTAB) Extraction

Cetyltrimethylammonium bromide extraction method is mainly used for plant samples and their parts, such as leaves, seeds, and grains. The method is used for various food samples as well. The basic composition of CTAB extraction buffer includes 2% CTAB at alkaline pH, but, like many other extraction protocols, CTAB has been modified according to the need of each sample [31]. CTAB works by precipitating nucleic acids and acidic polysaccharides in low ionic strength solutions, while proteins and neutral polysaccharides remain in solution. Next, the CTAB-nucleic acid precipitated complex is solubilized at high-salt concentrations, leaving the acid polysaccharides in the precipitate [1]. During the precipitation and washing steps, CTAB method uses various organic solvents and alcohols such as phenol, chloroform, isoamyl alcohol, and mercaptoethanol. The main drawback of this procedure is that it is time-consuming and makes use of toxic chemicals like phenol and chloroform. Moreover, CTAB extracted DNA requires further purification to avoid inhibition of PCR analyzes [32].

### 2.4. Chelex® Extraction

Chelex (Bio-Rad Laboratories, CA, USA) is a chelating resin frequently used in the field of forensics for DNA extraction from various sources, such as hair, blood stain cards, and buccal swabs [33]. According to [33], boiling in the presence of Chelex can increase the signal during PCR amplification of relatively minor amount of DNA, possibly by inhibiting DNA degradation by chelating metal ions which cause DNA breakdown at high temperature and lower ionic conditions. Chelex is a styrene divinylbenzene copolymer containing paired iminodiacetate ions, which are used as chelators for polyvalent metal ions [34]. This technique is interesting as it is quick, has few manipulating steps, and does not use hazardous chemicals such as phenol/chloroform. Its main drawback is the inability to efficiently remove PCR inhibitors from complex samples due to the lack of purification steps [35]. This method is also not suitable for restriction fragment length polymorphism (RFLP) analyses, because exposure of DNA to the high temperature and alkalinity of this protocol results in denaturation and breakage of DNA.

### 2.5. Alkaline Extraction

Alkaline extraction method is dedicated to plasmid DNA isolation, described by Bimboim and Doly [36]. The basic principle of this method is selective alkaline denaturation of high molecular weight chromosomal DNA, while covalently bond circular plasmid DNA remains intact. After neutralization, chromosomal DNA renatures and makes an insoluble precipitate, while plasmid DNA remains in the supernatant. This method is useful for both small and large DNA plasmids [36].

The method involves harvesting the bacteria of interest from culture media and exposing them to alkaline solution (consisting basically of SDS and NaOH). SDS act as detergent to lyse the cells and denature proteins, while alkaline condition denatures genomic DNA, plasmid DNA, and proteins. Potassium acetate (pH 5.2) addition neutralizes the mixture and results in renaturation of plasmid as well as genomic DNA. Further addition of ethanol (or isopropanol) precipitates genomic DNA, while plasmid DNA can be collected from the supernatant after a short 2-minute centrifugation. This technique is considered one of the fastest, most reliable, and relatively easy ways to obtain plasmid DNA from cells. Vigorous mixing during lysis and neutralization phases can cause fragmentation of genomic DNA, resulting in contamination with plasmid supernatant. The purified DNA is suitable for less sensitive applications. For more sensitive applications, a purifying step is needed, usually with spin columns.

### 2.6. Purification of Poly(A)+ RNA by Oligo(dT)-Cellulose Chromatography

Most of eukaryotic mRNA molecules possess a polyadenylated (polyA) tail of about 250 nucleotides at 3′ end. This provides foundation for a simple and easy way of RNA extraction through chromatographic techniques. The basic mechanism of this method is that poly(A) RNA hybridizes with an oligo(dT)-cellulose matrix, under high-salt conditions. Eukaryotic mRNAs have a diverse range in terms of size (from 0.5 kb to over 20 kb) and abundance (from fewer than 15 copies to over 20,000 copies per cell) [37]. Polyadenylated RNA with minimum 20 residues has the ability to attach to the oligo(dT)-cellulose matrix, which usually consists of 10–20 nucleotides [38]. After washing out all the nonpolyadenylated RNAs, a low-salt buffer is used to disrupt the oligo(dT)-poly(A) bond, resulting in the elution of poly(A) RNAs [39]. The selection for poly(A) RNA can be made in column or batch chromatography [1], being fast and yielding good RNA recovery. Its drawback resides in the fact that the method selects only mRNAs and naturally excludes important biological information present in other RNAs, such as miRNAs, rRNAs, and tRNAs.

Table 2 summarizes the main advantages and disadvantages of the chemically driven methods discussed here.

## 3. Solid-Phase Nucleic Acid Extraction

Solid-phase extraction (SPE) is one of the most efficient NAE techniques available in the market [1, 5]. It is based on liquid and stationary phases, which selectively separate the target analyte from the solution based on specific hydrophobic, polar, and/or ionic properties of both solute and sorbent. The chemistry between sorbent and analyte of interest is the basis of this technique, while “weak” chemical interactions such as van der Waals forces (nonpolar interactions), dipole-dipole interactions (polar interactions), and hydrogen bonding determine the retention mechanism in SPE.

SPE methods can be divided into normal/regular SPE, reverse SPE, and ion exchange SPE. Every sorbent used in SPE has unique characteristics, which give rise to a solution for a specific problem involved in extraction methods. A good example is acetonitrile, which decreases the polarity of the solution and decreases the interaction of DNA molecules with the stationary phase. Normally, reverse SPE uses polar/moderately mobile phase, nonpolar stationary phase, and semi- or nonpolar analytes, while normal SPE consists of semi- to nonpolar mobile phase, polar stationary phase, and polar analytes. On the other hand, ion exchange SPE is based on electrostatic interaction of both sorbent and the analyte of interest [40].

Solid-phase microextraction (SPME) is a relatively new development in solid-phase extraction technique, introduced in 1990s by [41], being useful for various analytes including liquid, gaseous, and solid matrices [42]. Two important steps are involved in SPME: (i) partitioning of analytes on fiber-coated extraction phase and (ii) handing over extract to separating instrument like gas chromatography where desorption takes place. SPME is a rapid and easy to use technique and have good detection limit (parts per trillion) for specific compounds [43]. Drawbacks of SPME include difficulty in analyzing high molecular weight compounds, sample carryover, and the eventual shortage of commercially available stationary phases.

### 3.1. Silica Matrices

In 1979, it was found that silicates have high binding affinity for DNA under alkaline conditions and increased salt concentration [44]. Silica matrices have revolutionized NAE procedures for both commercial as well as research purposes. Efficient and selective binding of NA to silica matrices is the hallmark of this fast and robust NA purification procedure [45]. Silica matrices consist of silica material, in the form of either gel or glass particle (i.e., glass microfibers) [46]. The mechanism involved in this technique is the affinity between negatively charged NA and positively charged silica material, resulting in selective binding of nucleic acids to the silica matrices, while the rest of the cell components and other chemicals are washed out. Silica surface is covered by positive ions, which enhances the binding of negatively charged DNA. As a final step, NA can be eluted from silica matrix by any hyposmotic solution, such as nuclease-free water or buffers such as alkaline Tris-EDTA. For RNA extraction, chaotropic agents have a second and very important task in denaturing RNases [47]. Many modifications have been made to the original procedure, such as introduction of hydrated silica matrix and microchip-based silica SPE [48]. In this technique, it is also noteworthy the role played by sodium ions in attracting the negatively charged oxygen present in nucleic acid's phosphate group and helping NA become insoluble because of the phenomenon known as “salting out” in the presence of high-salt conditions and acidic pH [4]. This technique provides high-purity DNA, is easy to perform, and also is able to reproduce quantitatively as well as qualitatively. Downside of this technique is being unable to recover small fragments DNA efficiently, as small fragments binds tightly with the silica matrix [49].

### 3.2. Glass Particles

Glass particles, whether in powder as chromatography stationary phase or in microbeads form, have also been used for extraction of nucleic acids. Chaotropic salts are used to release the NA and allow binding to common silicate glass, flint glass, and borosilicate glass (arranged as glass fiber filters). The basic principle of binding of NA to glass particles is based on the adsorption affinity of the components present in the mixture (DNA, proteins, etc.) to the stationary phase of chromatography column (glass particles). Polar compounds such as DNA have a high tendency of attachment to polar stationary phase under specific ionic strength [46].

### 3.3. Diatomaceous Earth

Diatomaceous earth (DE), alternatively known as kieselguhr or diatomite, is mainly composed of silica 80% to 90% (sometimes up to 95%), alumina 2% to 4%, and hematite 0.5% to 2% [50]. First described by Boom et al. [51], this procedure is mainly based on the binding of NA to a solid phase (such as DE) in the presence of chaotropic agents, followed by elution in water or low-salt buffer. NA binds to the silica present in DE, following the same principles of binding to silica matrices. This procedure has the advantage of reduced pipetting error, shorter protocol time, and less number of steps for sample preparation, being used for plasmid as well as for single or double-stranded nucleic acids [52]. However, this technique is not routinely used because of comparably high cost.

### 3.4. Magnetic Beads-Based Nucleic Acid Purification

Magnetic beads technology is one of the emerging strategies for extracting RNA and genomic, plasmid, and mitochondrial DNA. The technique involves the separation of nucleic acids from complex mixtures via complementary hybridization [53]. In recent years, functionalized magnetic particle or beads have been coupled to suitable buffers systems for a rapid and efficient extraction procedure [54]. The lack of centrifugation steps that can produce shear forces and cause breaking of nucleic acids is thought to better maintain intact longer fragments from genomic DNA. Usually, it is enough to apply a magnet to the side of a vessel or tube containing the sample mixed with the functionalized magnetic beads and exclusively aggregate the target particles near the vessel wall. The positive aspect of this technique is avoiding centrifugation steps as well as providing an alternative way for automation of extraction procedures from a large number of samples. The extraction technique can be used in batch processes with a multitude of samples (blood, tissues, and others) and is relatively easy to execute, being one of the best choices for automation, high-throughput applications, and high sample processivity [55, 56]. This method is also suitable for using in low technological environments because it is virtually equipment-free.

### 3.5. Anion Exchange Material

Just like silica matrices, anion exchange resins are also widely used in DNA and RNA extraction [57]. Unlike silicate negative charge, anion exchange resin makes use of the positively charged diethylaminoethyl cellulose (DEAE) to attract the negatively charged phosphate of nucleic acid. So, pH and salt concentration are the important aspects determining the binding or elution of NA to the anion exchange resin [58]. Anion exchange has the advantage of extracting very pure DNA as compared to silica and the ability to reuse the resin upon renaturation. However, this method used high-salt concentration in the elution step, thus requiring desalting for downstream applications.

### 3.6. Cellulose Matrix

Absorbent cellulose-based paper is an interesting matrix for nucleic acids purification and storage. Cellulose is a polymer of glucose and therefore highly hydroxylated, producing a polar attraction strong enough to bind nuclei acids under specific chemical conditions. The commercially available FTA paper (Whatman Inc., USA) or fast technology for analysis is an interesting example of how to purify and store DNA using cellulose. FTA cards are simply a thick layer of paper, embedded with a proprietary mix of buffers, detergents, and chelating agents, such as the ubiquitous Tris pH 8, SDS, and EDTA. Once cells are spotted onto the paper, the detergent lyses the membranes and EDTA chelates metal ions that are cofactors to nucleases, also preventing the growth of contaminating organisms [59, 60]. Hence, when dried, nucleic acids are relatively well protected from the environment, especially due to the unavailability of water molecules. In fact, DNA extraction from dried blood spotting has been successfully used for PCR-mediated human diagnostics for more than 20 years [61–63].

Although FTA cards have many advantages regarding the easiness of use and storage, processing them to extract good yields of nucleic acids might be more complicated than expected, especially in diluted samples [64].

Table 3 summarizes the main advantages and disadvantages of most commonly used solid-phase extraction methods.  Table 4 gives examples of commercially available kits using the methods described herein, as well as giving typical yields for NA extraction.

## 4. Devices Used in Extraction Methods

### 4.1. Spin Columns

The binding element in spin-column systems is usually composed of glass particles or powder, silica matrices, diatomaceous earth, and ion exchange carriers. Nucleic acid binding is thus optimized with specific buffer solutions and extremely precise pH and salt concentrations [4]. Column-based NAE is one of the best techniques among the options available, playing a vital role in ion exchange methods, as it provides a robust stationary phase for a rapid and reliable buffer exchange and thus NAE. This method is fast and reproducible, and its main drawback is the need for a small centrifuge as equipment requirement.

### 4.2. Beads or Magnetic Beads

Magnetic particle or beads are the first option to eliminate centrifuge-dependent steps in the extraction process. Magnetic beads make use of different ligands such as antibodies, antigens, oligonucleotides, or aptamers, which bind specifically to its target in sample. The first magnetic particle used for extraction consisted of an iron-oxide core covered by functional carboxylic group, which then binds DNA or RNA [81]. Since then, many modifications have been made using different surface functional group, such as sulphate, amino, and hydroxyl groups [82]. Besides these functional groups, preactivated magnetic beads with different functional groups are available such as tosyl or epoxy groups [78, 83]. Magnetic beads activated with protein A, protein G, or streptavidin are commercially available. Magnetic bead separation presents many benefits over centrifuge-dependent extraction process by allowing an equipment-free process. Equipment-free separation of NA is also possible with nonmagnetic beads, where the beads are trapped inside plastic bubble pipets [84].

### 4.3. Automation (Liquid Handling Robots)

The increase in growth of diagnostic tests and patient numbers highlights the need for automation in life sciences [85]. To fulfill this demand, various automated devices have been developed and introduced in the market. The most successful examples are the automated liquid handling robots, which are routinely used in many life science and clinical analysis laboratories for dispensing precise amount of sample, reagents, or other liquids to designated containers. Because of this technology, it is now possible to handle many samples simultaneously with precision and rapidity. In addition, barcode readers are an integral part of such equipment, allowing for easy traceability of samples and results. Fully automated NAE protocols have been developed for such equipment, using either solid-phase or magnetic beads methods [79]. However, high sample processivity is a positive aspect of automation while maintaining the sensitivity can be compromised, as low-copy NA targets might be lost [86]. Small versions of these robots are available and could be useful in laboratory settings with minimal infrastructure. Liquid handling robots certainly have a niche in life sciences and clinical laboratories, but not as POC devices.

### 4.4. Microfluidics and “Lab-on-a-Chip” Cartridges

Nucleic acid-based detection (NAT) is preferable compared to immunoassay-based detection because of sensitivity and specificity, but NAT-based diagnosis requires complex infrastructure, sophisticated machinery, and trained personnel. To overcome this hurdle, microfluidic chips have been designed and produced, carrying, on inner chambers, all necessary reagents for molecular based tests as a part of POC-Dx strategy. Usually, microfluidic chips (or “lab-on-a-chip” cartridges) rely on solid phase extraction for NA isolation, in the form of either membranes or beads [44, 87].

The union of automation with the need for miniaturization in POC devices led to the development of cartridges that perform one or several biological reactions in a closed container. These reactions comprise most of the current molecular biology methods, such as NAE, amplification, and identification, as well as serological signatures analyses. One of the greatest examples of a microfluidic cartridge, although not POC, is the milestone related to diagnosis of* Mycobacterium tuberculosis* (Mtb) achieved by the platform GeneXpert MTB/RIF [80]. Specific cells/spores are selected through filtration followed by a lysing step (sonification). Microtubes, pumps, and rotary drives transfer liquids into the specific cartridge chambers where washing, purification, and concentration of nucleic acids take place. The next step is the movement of the extracted NA to a reaction chamber where real-time PCR happens [88]. A recent systematic meta-analysis study reviewed hundreds of papers concluded that GeneXpert was the most cost-effective strategy for POC-Dx of Mtb, although its performance was evaluated solely in clinics and primary care centers [89]. However, it is undisputed that GeneXpert is a breakthrough in NA testing.

The FilmArray 2.0 system (BioFire Diagnostics LLC, Salt Lake City, USA) is a multiplexed PCR system that incorporates specimen processing, NA amplification, and detection in a specialized pouch. Specific pouches are used to amplify different targets present in the sample, using Nested PCR, followed by real-time PCR with chemistry-based detection. The software then automatically generates identification reports using DNA melting analysis based on specific control reactions or a melting curve database of known sequences.

Table 5 presents a summary of the devices available most commonly used in NAE protocols.

## 5. Limitations for Implementation of Extraction Protocols in Portable Devices

A major obstruction for the development of a complete and easy-to-use solution for POC-Dx is the integration of sample preparation protocols into the portable devices. Removing interferents and extracting the target molecules are no trivial task especially due to the vast differences among sample matrices as well as characteristics of the target analytes. While NAE protocols are well established in the laboratory and many advances have been made since the inception of microfluidic Dx devices, commercial availability of these devices is still rare [90]. Excellent reviews are available discussing the technical difficulties as well as the obstacles for implementation and acceptance of new tests based on new technologies [90–94].

Several organic chemicals routinely used in molecular biology can react with the plastic materials commonly used in POC cartridges/devices, which makes difficult for some polymers to sustain their initial mechanical and physicochemical properties. One of properties paramount to the performance characteristics of the plastic materials is chemical inertness, that is, the material to which the active substance of interest will be in contact with will not interact and generate undesirable products, generally classified as extractable or leachable [95]. Toxicological or functional studies often replace extraction and interaction studies, which would be necessary to determine the levels of extractable or leachable products under a given environmental condition. Such replacement is acceptable, although not ideal, because the biological assessment performed for toxicological studies should include basic extraction/interaction evaluations [95]. Studies of structural properties of glassy polymers such as the commonly used thermoplastics polycarbonate (PC) and polymethylmethacrylate (PMMA) correlate the polymer solubility when exposed to several solvents to the extent of stress cracking [96]. An advantage of PMMA is its high optical transparency into the ultraviolet range, while PC offers a compatibility with a wider range of solvents and a higher glass transition temperature well suited to applications such as polymerase chain reaction for NA amplification [97]. However, neither of these is good enough to be used with the chemicals routinely used for NAE. For example, PMMA cannot be cleaned by strong solvents such as acetone or methanol, because these chemicals would significantly damage its surface and decrease transparency [97] (Table 6).

Some chemicals have the potential to affect polymer's color, surface appearance, flexibility, and mass, generating extractable/leachable products that must be evaluated. These changes can happen due to several physicochemical reactions, such as (i) chemical interaction with polymer chain which can disturb their structure and result in depolymerization; (ii) physical interaction, that is, adsorption of chemicals into the plastics, which results in swelling and softening; or (iii) stress-associated cracking may happen due to the stress-cracking agents, such as plasticizers, or adhesives used during the manufacturing of polymer parts, or even detergents or oils used during the molecular biology processes [98]. Table 6 lists the effect of the chemicals most commonly used in NAE on the plastics most commonly used for microfabrication of microdevices. Alterations induced by any chemical, as minor as it seems, need to be thoroughly evaluated. In extreme cases, chemicals must be substituted, such as that of ethanol/isopropanol. Ethanol/isopropanol storage in cartridges is also problematic because of its volatility, flammability, and potential to leak. Such chemical properties make alcohols a substance highly regulated by the International Air Transport Association (IATA). Therefore, substitutes such as diethylene glycol monoethyl ether (DGME) and diethylene glycol monoethyl ether acetate (DGMEA) [99], must be tested and validated.

Finally yet importantly, there is concern about the volume of sample needed to obtain a meaningful results [100]. Because the volume of buffers and, therefore, of harsh chemicals used for cell lysis is directly proportional to the volume of the sample, POC-Dx tests are most useful in illness where the pathogen is present in higher counts, such as virus and most bacterial infections. Parasitic infections, however, present a challenge to POC-Dx because parasite loads can get very close to the limits of detection of the techniques used [101], thus greatly affecting the availability of target NA in the sample. The volume of the reagents is also important to assure proper mixing of solutions without the common laboratory instruments because small volumes are easier to homogenize [102].

## 6. Challenges for Implementation in POC Diagnostic Tests

Lessons learned from previous attempts in developing diagnostic tests have taught us that availability of the best possible POC-Dx test is not enough. Its implementation is also very important and often underestimated, since only few diseases have a validated POC-Dx, such as HIV or malaria [103, 104]. Implementation should be considered during the development phase of the POC-Dx, so that end-users are identified, their level of experience is assessed, and the developing test is used at the right lab tier [92].

The major features of POC tests are described by the WHO acronym ASSURED: affordable, sensitive, specific, user-friendly, equipment-free, and deliverable to end-users [103]. The main idea is to provide low cost and timely effective healthcare to the patient and quick decision making for healthcare providers. One platform which seems to have the potential to meet the ASSURED criteria is microfluidic paper-based analytical devices (*µ*PADs), which provide a robust, affordable, equipment-free, and multiplex facility [105–107]. Paper-based devices are abundant, either directly operating or directing the biochemical, serological, or nucleic acid reactions [106, 107]. Because they are easily manipulated to attach recognition molecules (antibodies, enzymes, proteins, nucleic acids, etc.), *µ*PADs devices have been very successful in several areas of biological research, such as biochemical analysis of blood or urine, detection of pathogen's nucleic acids, detection of drugs, or environmental contamination. *µ*PADs can also be designed for direct sensing the target molecule by using nanotechnologies, such as microelectromechanical systems, field effector transistors, or nanocantilevers. However, since describing each of the available *µ*PADs is beyond the scope of our review, the reader is directed to other available texts on the subject [106–109]. Unlike protein or metabolite-based POC tests, one of the major challenges for nucleic acid-based POC tests is the need to consolidate three distinct protocol procedures into a single device: (1) nucleic acid extraction; (2) amplification; and (3) detection. Development of a nucleic-based testing device that is specific, sensitive, portable, and relatively easy to operate has presented several challenges that have been elegantly reviewed elsewhere [90]. Below we discuss the challenges strictly related to NAE for POC-Dx.

Development of an ideal NAE method for POC is impaired by many factors and researchers are still in quest for a suitable solution. At present, solid-phase extraction [110] and magnetic beads [54] are the primary choices for NAE in POC-Dx devices. However, neither method is yet good enough for widespread implementation in POC-Dx methods. Solid-phase extraction depends on centrifugation, while magnetic beads require an external magnet source for mixing. In this aspect, magnetic beads are favored because implementation of magnetic stirring in POC-Dx devices is somewhat easier than implementation of separation through stationary membranes. Although both rely on the use of chaotropic reagents for lysing cells and releasing the NA from the scaffold and structural nucleic proteins, washing steps are more efficient in beads-based methods. The main challenges in implementing molecular biology-based systems in resource-constrained areas are the high cost of instrument and reagents, as well as lack of reliable infrastructure and continuous maintenance support and temperature maintenance devices [88]. Proper disposal of biological waste generated by medical tests is also a concern, not to mention that some waste is chemical and requires special treatment before disposal (e.g., guanidine thiocyanate) [92].

## 7. Conclusion

After almost 150 years after the first successful isolation of DNA by Friedrich Miescher, nucleic acids are now central to obtaining biological information in areas as distinct as specimens' identification for conservational purposes to the realms of personalized medicine and pharmacogenomics. Protocols and devices used for NAE have evolved from thiocyanate-phenol-chloroform manual techniques to user-friendly column-technology and automated platforms, but no general gold-standard method has yet been established. This review analyzed the working principle of each available method, as well as their advantages and disadvantages. The take-home message is that each application has specific characteristics, which should then guide each researcher to the most suitable method.

Although molecular biology techniques are sensitive and accurate methods, they require a rather well established laboratory setting and expensive instruments, as well as skilled personnel to run the tests and analyze the results, which are not always available. In the last years, lab-on-chip technology has brought the promise of taking the management of biological information where it is needed, such as low-resource settings, a doctor's clinic or a hospital patient bedside. However, although progress has been made, several obstacles still hamper the use of NAE protocols in POC-Dx tests, as it can be seen by the low number of products using lab-on-chip technology. Overcoming the challenges and limitations of NAE protocols will greatly increase the use of molecular biology techniques and thus increase the overall quality of life of the general population by providing access to better diagnostic tests.

## Figures and Tables

**Table 1:** Main characteristics of chemical and mechanical methods to extract nucleic acid (adapted from Harrison 2003).

Method	Technique	Principle	Mode of lysis	Cost	Most usual application	References
Chemical	Osmotic shock	Osmotic rupture of membrane	Gentle	Cheap	Spheroplasts and Protoplasts	[9]
	Enzymatic digestion	Digestion of cell wall	Gentle	Cheap at small scale; expensive at large scale	Gram-positive and Gram-negative bacteria	[13]
	Detergents	Solubilization of membranes	Gentle	Moderate	General use	[14]
	Alkali treatment	Solubilization of membrane	Harsh	Cheap	Plasmid DNA	[15]

Mechanical	Homogenization (blade or pestle)	Shredding of cells	Moderate	Moderate (method of choice for large scale)	Animal tissues	[10]
	Ultrasonication or cavitation	Disruption of cells by pressure	Harsh	Moderate to expensive	Good for spheroplasts but not primary cells	[11, 12]
	Pressure cell (“French press”)	Disruption of cells by shear force	Harsh	Moderate	Used for Gram-negative and some Gram-positive bacteria	[6]
	Ball mill	Cells crushed between glass/steel balls/beads	Harsh	Cheap	Used for bacteria, yeast, microalgae, unicellular animal cells	[15]

**Table 2:** Summary of advantages and disadvantages of the main NAE methods. GuSCN, guanidine thiocyanate; CsCl, cesium chloride; EtBr, ethidium bromide; CTAB, cetyltrimethylammonium bromide.

Method	Advantage	Disadvantage	Reference
(1) GuSCN-phenol- chloroform extraction	High purity and yield of DNA or RNA	Hazardous chemicals	[21, 23]
(2) Alkaline extraction	Fastest, reliable, and relatively easy procedure	Medium purity and fragmentation of genomic DNA	[26]
(3) CsCl gradient centrifugation with EtBr	High purity and yield of DNA or RNA	Laborious, costly and time consuming,	[29, 30]
(4) Oligo(dT) cellulose chromatography	Fast protocol, good yield of mRNA recovery	Purification bias for mRNAs	[1]
(5) Chelex® extraction	Quick and simple protocol; no use of hazardous chemicals	Low purity of nucleic acids	[35, 36]
(6) CTAB extraction	Efficient method for plant and other “hard to lyse” samples	Laborious, time-consuming; use of hazardous chemicals	[38]

**Table 3:** Summary of the advantages and disadvantages of solid-phase extraction methods.

Material	Molecule of affinity	Advantage	Disadvantage	Reference
(1) Silica matrices	DNA, RNA	High-purity DNA, easy to perform, and reproducible	Unable to recover small DNA fragments; one-time use	[52]
(2) Glass particles	DNA, protein	Simple, sensitive, and reproducible	High cost; requirement of equipment	[1, 52]
(3) Diatomaceous earth	DNA, RNA	Reduced pipetting error, shorter protocol (less time and steps)	High cost	[53]
(4) Magnetic beads	DNA, RNA	No centrifugation, best choice for automation, virtually equipment-free	Interference in PCR amplification	[56]
(5) Anion exchange material	DNA, RNA	Reusable resins	Presence of high-salt concentrations	[58]
(6) Cellulose matrix	DNA, RNA	Easy to use and storage	Extraction protocols being complex and prone to error	[60]

**Table 4:** Examples of commercially available kits applying each extraction method and typical yields for distinct samples.

Method	Commercial availability	Sample origin	Typical yield	Reference
GSCN-phenol-chloroform extraction	TRIzol reagent (e.g.,, Invitrogen)	Mammalian cells (10^6^ cells)	Epithelial cells, 8–15 *µ*g; fibroblasts, 5–7 *µ*g.	[65]
Alkaline extraction	Plasmid Maxi Kit (e.g., Qiagen)	Cultured bacteria (2.5 L)	Up to 500 *µ*g of plasmid DNA	[66–68]
CTAB	NucleoSpin 8 Plant and NucleoSpin 96 Plant II (e.g., Clontech)	Plant material 20–100 mg plant tissue (wet weight)	1–30 *µ*g	[69, 70]
Silica matrices	QIAamp DNA mini kit (e.g., Qiagen)	Blood (200 *µ*l), buffy coat (200 *µ*l) or 10^6^ cells	4–12 *µ*g DNA (blood) 25–50 *µ*g DNA (buffy coat) 15–20 *µ*g DNA (cells)	[71, 72]
Magnetic bead	Agencourt DNAdvance Kit (e.g., Beckman Coulter)	Mammalian tissues (25 mg of sample)	18–35 *µ*g DNA	[73]
Anion exchange material	PureLink® HiPure Plasmid DNA Purification Kits (e.g., Invitrogen)	Culture bacteria: midipreps (15–25 mL) or maxiprep (up to 200 mL)	350 *µ*g for midipreps 850 *µ*g for maxipreps	[74]
Diatomaceous Earth	Quantum Prep Plasmid Purification Kits (e.g., Bio-Rad)	Culture bacteria (1-2 mL liquid culture)	Up to 40 *µ*g	[75]
Cellulose matrix	FTA cards (e.g., Whatman)	8 × 2 mm punches	1–5 *µ*g (plant) 1–3 *µ*g (dried blood spots)	[61–63, 76, 77]

**Table 5:** Summary of available devices used in nucleic acid extraction protocols.

Material	Molecule of affinity	Advantage	Disadvantage	Reference
(1) Spin columns	DNA, RNA	Fast; reproducible	Aerosols cross-contamination; infrastructure and equipment required	[49]
(2) Beads or magnetic beads	DNA, RNA	Simple to use; high automation potential; equipment-free process	Labor intensive	[78]
(3) Automation (liquid handling robots)	DNA, RNA	Precise manipulation of sample and reagents, reducing losses and cost	High cost	[79]
(4) Microfluidics and “lab-on-a-chip” cartridges	DNA, RNA	Sensitive and specific	Incompatibility of common NAE chemicals	[80]

**Table 6:** Chemical compatibility of various chemicals used in nucleic acid extraction procedures and plastic polymers commonly used in microfabrication. (PDMS, polydimethylsiloxane; PMMA, polymethylmethacrylate; PS, polystyrene; PC, polycarbonates.) Information was gathered from multiple sources, such as the following vendor's websites: http://www.permselect.com, http://www.labicom.com.cz, https://www.coleparmer.co.uk/Chemical-Resistance.

Chemicals∖Plastic polymer	PDMS	PMMA	PS	PC
Ethanol	Good	Good	Not compatible	Excellent
Phenol 10%	Not compatible	Not compatible	Not compatible	Not compatible
Chloroform	Not compatible	Not compatible	Not compatible	Not compatible
Detergents	Excellent	—	—	—
Urea	Good	Excellent	Excellent	Excellent
Guanidinium thiocyanate	—	Good	—	Excellent
Methyl alcohol	Excellent	Good	Good	Fair
Alcohols: isopropyl	Excellent	Excellent	Excellent	Fair
Alcohols: ethyl	Good	Good	Good	Excellent
Alcohols: amyl	Not compatible	Excellent	Good	Good
HCl 37%	Not compatible	—	—	—
HCl 20%	Good	Good	Good	Good

## References

[1] Tan S. C., Yiap B. C. (2009). DNA, RNA, and protein extraction: the past and the present. *Journal of Biomedicine and Biotechnology*.

[2] Doyle K. (1996). *The Source of Discovery: Protocols and Applications Guide, PROMEGA*.

[3] Lesk A. M. (1969). Why does DNA contain thymine and RNA uracil?. *Journal of Theoretical Biology*.

[4] Chacon-cortes D., Griffiths L., Chacon-Cortes D. (2014). Methods for extracting genomic DNA from whole blood samples: current perspectives. *Journal of Biorepository Science for Applied Medicine*.

[5] Price C. W., Leslie D. C., Landers J. P. (2009). Nucleic acid extraction techniques and application to the microchip. *Lab on a Chip—Miniaturisation for Chemistry and Biology*.

[6] Goldberg S. (2008). Mechanical/physical methods of cell distribution and tissue homogenization. *Methods in Molecular Biology*.

[7] Mitra S., Winefordner J. D. (2003). Sample preparation techniques in analytical chemistry. *Chemical Analysis - A Series of Monographs on Analytical Chemistry and Its Applications*.

[8] Burden D. (2012). Guide to the Disruption of Biological Samples. *Random Prim*.

[9] Kaya G, Dale C., Maudlin I., Morgan K. (2007). A novel procedure for total nucleic acid extractionfrom small numbers of eimeria species oocysts. *The Turkish Society for Parasitology*.

[10] Peternel Š., Komel R. (2010). Isolation of biologically active nanomaterial (inclusion bodies) from bacterial cells. *Microbial Cell Factories*.

[11] Rodríguez-Carmona E., Cano-Garrido O., Seras-Franzoso J., Villaverde A., García-Fruitós E. (2010). Isolation of cell-free bacterial inclusion bodies. *Microbial Cell Factories*.

[12] Prabakaran P., Ravindran A. D. (2011). A comparative study on effective cell disruption methods for lipid extraction from microalgae. *Letters in Applied Microbiology*.

[13] Salazar O., Asenjo J. A. (2007). Enzymatic lysis of microbial cells. *Biotechnology Letters*.

[14] Flahaut S., Frere J., Boutibonnes P., Auffray Y. (1996). Comparison of the bile salts and sodium dodecyl sulfate stress responses in Enterococcus faecalis. *Applied and Environmental Microbiology*.

[15] Harrison R. G., Todd P., Rudge S. R., Petrides D. P. (2003). *Bioseparations Science and Engineering*.

[16] Liu D., Zeng X.-A., Sun D.-W., Han Z. (2013). Disruption and protein release by ultrasonication of yeast cells. *Innovative Food Science and Emerging Technologies*.

[17] Klimek-Ochab M., Brzezińska-Rodak M., Zymańczyk-Duda E., Lejczak B., Kafarski P. (2011). Comparative study of fungal cell disruption-scope and limitations of the methods. *Folia Microbiologica*.

[18] Greenly J. M., Tester J. W. (2015). Ultrasonic cavitation for disruption of microalgae. *Bioresource Technology*.

[19] Bunge F., Pietzsch M., Müller R., Syldatk C. (1992). Mechanical disruption of Arthrobacter sp. DSM 3747 in stirred ball mills for the release of hydantoin-cleaving enzymes. *Chemical Engineering Science*.

[20] Brown T. A. (2010). *Gene cloning & DNA Analysis An Introduction Sixth edition*.

[21] Cseke L. J., Kaufman P. B., Kirakosyan A., Westfall M. V. Molecular and Cellular Methods in Biology and Medicine.

[22] Carr S. M., Griffith O. M. (1987). Rapid isolation of animal mitochondrial DNA in a small fixed-angle rotor at ultrahigh speed. *Biochemical Genetics*.

[23] Nath K., Sarosy J. W., Hahn J., Di Como C. J. (2000). Effects of ethidium bromide and SYBR(®) Green I on different polymerase chain reaction systems. *Journal of Biochemical and Biophysical Methods*.

[24] Ohta T., Tokishita S.-I., Yamagata H. (2001). Ethidium bromide and SYBR Green I enhance the genotoxicity of UV-irradiation and chemical mutagens in E. coli. *Mutation Research—Genetic Toxicology and Environmental Mutagenesis*.

[25] Chomczynski P., Sacchi N. (1987). Single-step method of RNA isolation by acid guanidinium thiocyanate-phenol-chloroform extraction. *Analytical Biochemistry*.

[26] Ullrich A., Shine J., Chirgwin J. (1977). Rat insulin genes: construction of plasmids containing the coding sequences. *Science*.

[27] Chirgwin J. M., Przybyla A. E., MacDonald R. J., Rutter W. J. (1979). Isolation of biologically active ribonucleic acid from sources enriched in ribonuclease. *Biochemistry*.

[28] Chomczynski P., Sacchi N. (2006). The single-step method of RNA isolation by acid guanidinium thiocyanate-phenol-chloroform extraction: twenty-something years on. *Nature Protocols*.

[29] Meng L., Feldman L. (2010). A rapid TRIzol-based two-step method for DNA-free RNA extraction from Arabidopsis siliques and dry seeds. *Biotechnology Journal*.

[30] Murnane M. J., Tam S. W. (1992). Isolation and characterization of RNA from snap-frozen tissues and cultured cells. *The Journal of Nutritional Biochemistry*.

[31] Terry C. F., Harris N., Parkes H. C. (2002). Detection of genetically modified crops and their derivatives: critical steps in sample preparation and extraction. *Journal of AOAC International*.

[32] Demeke T., Jenkins G. R. (2010). Influence of DNA extraction methods, PCR inhibitors and quantification methods on real-time PCR assay of biotechnology-derived traits. *Analytical and Bioanalytical Chemistry*.

[33] Walsh P. S., Metzger D. A., Higuchi R. (1991). Chelex 100 as a medium for simple extraction of DNA for PCR-based typing from forensic material. *BioTechniques*.

[34] Phillips K., McCallum N., Welch L. (2012). A comparison of methods for forensic DNA extraction: Chelex-100® and the QIAGEN DNA Investigator Kit (manual and automated). *Forensic Science International: Genetics*.

[35] Schrader C., Schielke A., Ellerbroek L., Johne R. (2012). PCR inhibitors—occurrence, properties and removal. *Journal of Applied Microbiology*.

[36] Bimboim H. C., Doly J. (1979). A rapid alkaline extraction procedure for screening recombinant plasmid DNA. *Nucleic Acids Research*.

[37] Bryant S., Manning D. L. (2000). Isolation of mRNA by affinity chromatography. *The Nucleic Acid Protocols Handbook*.

[38] Walker J. M. (1984). *Nucleic Acids*.

[39] Sambrook J., Russel W. (2000). *Molecular Cloning, 3-Volume Set: A Laboratory Manual*.

[40] Biziuk M. (2006). Solid Phase Extraction Technique—Trends. *Polish Journal of Environmental Studies*.

[41] Arthur C. L., Pawliszyn J. (1990). Solid phase microextraction with thermal desorption using fused silica optical fibers. *Analytical Chemistry*.

[42] Merkle S., Kleeberg K., Fritsche J. (2015). Recent developments and applications of solid phase microextraction (SPME) in food and environmental analysis—a review. *Chromatography*.

[43] Nielsen A. T., Jonsson S. (2002). Trace determination of volatile sulfur compounds by solid-phase microextraction and GC-MS. *Analyst*.

[44] Vogelstein B., Gillespie D. (1979). Preparative and analytical purification of DNA from agarose.. *Proceedings of the National Academy of Sciences*.

[45] Esser K.-H., Marx W. H., Lisowsky T. (2006). maxXbond: First regeneration system for DNA binding silica matrices. *Nature Methods*.

[46] V Padhye V., York C., Adam B. (1997). Nucleic acid purification on silica gel and glass mixture. *United States patent US 5658548*.

[47] Mutin A. I., Christopher J. B. (2005). RNA isolation from yeast using silica matrices. *Journal of Biomolecular Techniques*.

[48] Christel L. A., Petersen K., McMillan W., Northrup M. A. (1999). Rapid, automated nuleic acid probe assays using silicon microstructures for nucleic acid concentration. *Journal of Biomechanical Engineering*.

[49] Green M. R., Sambrook J. (2012). *Molecular Cloning*.

[50] Antonides L. E. Diatomite.

[51] Boom R., Sol C. J., Salimans M. M., Jansen C. L., Wertheim-van Dillen P. M., van der Noordaa J. (1990). Rapid and simple method for purification of nucleic acids. *Journal of Clinical Microbiology*.

[52] Koo K., Foegeding P. M., Swaisgood H. E. (1998). Isolation of RNA and DNA fragments using diatomaceous earth. *Biotechnology Techniques*.

[53] Archer M. J., Lin B., Wang Z., Stenger D. A. (2006). Magnetic bead-based solid phase for selective extraction of genomic DNA. *Analytical Biochemistry*.

[54] Azimi S. M., Nixon G., Ahern J., Balachandran W. (2011). A magnetic bead-based DNA extraction and purification microfluidic device. *Microfluidics and Nanofluidics*.

[55] Berensmeier S. (2006). Magnetic particles for the separation and purification of nucleic acids. *Applied Microbiology and Biotechnology*.

[56] Franzreb M., Siemann-Herzberg M., Hobley T. J., Thomas O. R. T. (2006). Protein purification using magnetic adsorbent particles. *Applied Microbiology and Biotechnology*.

[57] Seligson D. B., Shrawder E. J. (1995). *United States Patent [19]1 Patent Number: 5,387,720*.

[58] Qiagen Genomic DNA Handbook.

[59] Smith L. M., Burgoyne L. A. (2004). Collecting, archiving and processing DNA from wildlife samples using FTA® databasing paper. *BMC Ecology*.

[60] Burgoyne L. A. (1996). Solid medium and method for DNA storage.

[61] Cassol S. A., Lapointe N., Salas T. (1992). Diagnosis of vertical HIV-1 transmission using the polymerase chain reaction and dried blood spot specimens. *Journal of Acquired Immune Deficiency Syndromes*.

[62] Cassol S. A., Read S., Weniger B. G. (1996). Dried blood spots collected on filter paper: an international resource for the diagnosis and genetic characterization of human immunodeficiency virus type-1. *Memórias do Instituto Oswaldo Cruz*.

[63] Choi E.-H., Lee S. K., Ihm C., Sohn Y.-H. (2014). Rapid DNA extraction from dried blood spots on filter paper: potential applications in biobanking. *Osong Public Health and Research Perspectives*.

[64] Milne E., Van Bockxmeer F. M., Robertson L. (2006). Buccal DNA collection: comparison of buccal swabs with FTA cards. *Cancer Epidemiology Biomarkers and Prevention*.

[65] Hummon A. B., Lim S. R., Difilippantonio M. J., Ried T. (2007). Isolation and solubilization of proteins after TRIzol® extraction of RNA and DNA from patient material following prolonged storage. *BioTechniques*.

[66] Somvanshi V. S., Sloup R. E., Crawford J. M. (2012). A single promoter inversion switches photorhabdus between pathogenic and mutualistic states. *Science*.

[67] Klinge S., Voigts-Hoffmann F., Leibundgut M., Arpagaus S., Ban N. (2011). Crystal structure of the eukaryotic 60S ribosomal subunit in complex with initiation factor 6. *Science*.

[68] Antonic A., Sena E. S., Lees J. S. (2013). Stem cell transplantation in traumatic spinal cord injury: a systematic review and meta-analysis of animal studies. *PLoS Biology*.

[69] Niewiadomska A. M., Gifford R. J. (2013). The extraordinary evolutionary history of the reticuloendotheliosis viruses. *PLoS Biology*.

[70] Varenhorst C., James S., Erlinge D. (2009). Genetic variation of CYP2C19 affects both pharmacokinetic and pharmacodynamic responses to clopidogrel but not prasugrel in aspirin-treated patients with coronary artery disease. *European Heart Journal*.

[71] Rulli S. J., Mirro J., Hill S. A. (2008). Interactions of murine APOBEC3 and human APOBEC3G with murine leukemia viruses. *Journal of Virology*.

[72] Ogino S., Kawasaki T., Nosho K. (2008). LINE-1 hypomethylation is inversely associated with microsatellite instability and CpG island methylator phenotype in colorectal cancer. *International Journal of Cancer*.

[73] Fischer M. G., Suttle C. A. (2011). A virophage at the origin of large DNA transposons. *Science*.

[74] Nie H., Wang C.-H. (2007). Fabrication and characterization of PLGA/HAp composite scaffolds for delivery of BMP-2 plasmid DNA. *Journal of Controlled Release*.

[75] Johnston N. J., De Azavedo J. C., Kellner J. D., Low D. E. (1998). Prevalence and characterization of the mechanisms of macrolide, lincosamide, and streptogramin resistance in isolates of Streptococcus pneumoniae. *Antimicrobial Agents and Chemotherapy*.

[76] Siegel C. S., Stevenson F. O., Zimmer E. A. (2017). Evaluation and comparison of FTA Card and CTAB DNA extraction methods for non-agricultural taxa. *Applications in Plant Sciences*.

[77] Molteni C. G., Terranova L., Zampiero A., Galeone C., Principi N., Esposito S. (2013). Comparison of manual methods of extracting genomic DNA from dried blood spots collected on different cards: Implications for clinical practice. *International Journal of Immunopathology and Pharmacology*.

[78] Bruno J. G., Kiel J. L. (2002). Use of magnetic beads in selection and detection of biotoxin aptamers by electrochemiluminescence and enzymatic methods. *BioTechniques*.

[79] Rule G., Chapple M., Henion J. (2001). A 384-well solid-phase extraction for LC/MS/MS determination of methotrexate and its 7-hydroxy metabolite in human urine and plasma. *Analytical Chemistry*.

[80] Boehme C. C., Nabeta P., Hillemann D. (2010). Rapid molecular detection of tuberculosis and rifampin resistance. *The New England Journal of Medicine*.

[81] Hawkins T. DNA Purification and Isolation using magnetic particles.

[82] Adams N. M., Bordelon H., Wang K.-K. A., Albert L. E., Wright D. W., Haselton F. R. (2015). Comparison of three magnetic bead surface functionalities for RNA extraction and detection. *ACS Applied Materials and Interfaces*.

[83] Centi S., Laschi S., Mascini M. (2009). Strategies for electrochemical detection in immunochemistry. *Bioanalysis*.

[84] Pawlowski D. R., Karalus R. J. (2012). Electricity-free, sequential nucleic acid and protein isolation. *Journal of Visualized Experiments*.

[85] Markin R. S., Whalen S. A. (2000). Laboratory automation: trajectory, technology, and tactics. *Clinical Chemistry*.

[86] Thatcher S. A. (2015). DNA/RNA preparation for molecular detection. *Clinical Chemistry*.

[87] Mauk M. G., Liu C., Sadik M., Bau H. H. (2015). Microfluidic devices for nucleic acid (NA) isolation, isothermal NA amplification, and real-time detection. *Mobile Health Technologies: Methods and Protocols*.

[88] Dineva M. A., Mahilum-Tapay L., Lee H. (2007). Sample preparation: a challenge in the development of point-of-care nucleic acid-based assays for resource-limited settings. *Analyst*.

[89] Drobniewski F., Cooke M., Jordan J. (2015). Systematic review, meta-analysis and economic modelling of molecular diagnostic tests for antibiotic resistance in tuberculosis. *HealtH Technology Assessment*.

[90] Cui F., Rhee M., Singh A., Tripathi A. (2015). Microfluidic sample preparation for medical diagnostics. *Annual Review of Biomedical Engineering*.

[91] Huckle D. (2015). The impact of new trends in POCTs for companion diagnostics, non-invasive testing and molecular diagnostics. *Expert Review of Molecular Diagnostics*.

[92] Wang S., Lifson M. A., Inci F., Liang L.-G., Sheng Y.-F., Demirci U. (2016). Advances in addressing technical challenges of point-of-care diagnostics in resource-limited settings. *Expert Review of Molecular Diagnostics*.

[93] Hardy V., Thompson M., Alto W. (2016). Exploring the barriers and facilitators to use of point of care tests in family medicine clinics in the United States. *BMC Family Practice*.

[94] Busin V., Wells B., Kersaudy-Kerhoas M., Shu W., Burgess S. T. G. (2016). Opportunities and challenges for the application of microfluidic technologies in point-of-care veterinary diagnostics. *Molecular and Cellular Probes*.

[95] Jenke D. (2007). Evaluation of the chemical compatibility of plastic contact materials and pharmaceutical products; safety considerations related to extractables and leachables. *Journal of Pharmaceutical Sciences*.

[96] Bernier G. A., Kambour R. P. (1968). The role of organic agents in the stress crazing and cracking of poly(2,6-dimethyl-1,4-phenylene oxide). *Macromolecules*.

[97] Yang S., DeVoe D. L. (2013). Micro fluidic device fabrication by thermoplastic hot-embossing. *Methods in Molecular Biology*.

[98] Robeson L. M. (2013). Environmental stress cracking: a review. *Polymer Engineering and Science*.

[99] Himmelreich R., Werner S. Methods for Isolation and purifying nucleic acids.

[100] Fong E. J., Huang C., Hamilton J. (2015). A microfluidic platform for precision small-volume sample processing and its use to size separate biological particles with an acoustic microdevice. *Journal of Visualized Experiments*.

[101] Warkiani M. E., Tay A. K., Khoo B. L., Xiaofeng X., Han J., Lim C. T. (2015). Malaria detection using inertial microfluidics. *Lab Chip*.

[102] Weibel D. B., Whitesides G. M. (2006). Applications of microfluidics in chemical biology. *Current Opinion in Chemical Biology*.

[103] Wu G., Zaman M. H. (2012). Low-cost tools for diagnosing and monitoring HIV infection in low-resource settings. *Bulletin of the World Health Organization*.

[104] Glendon L. Horizon Scan 2015.

[105] Martinez A. W., Phillips S. T., Whitesides G. M., Carrilho E. (2010). Diagnostics for the developing world: microfluidic paper-based analytical devices. *Analytical Chemistry*.

[106] Yamada K., Shibata H., Suzuki K., Citterio D. (2017). Toward practical application of paper-based microfluidics for medical diagnostics: state-of-the-art and challenges. *Lab Chip*.

[107] Hu J., Wang S., Wang L. (2014). Advances in paper-based point-of-care diagnostics. *Biosensors and Bioelectronics*.

[108] Liu S., Su W., Ding X. (2016). A Review on Microfluidic Paper-Based Analytical Devices for Glucose Detection. *Sensors*.

[109] Busa L. S. A., Mohammadi S., Maeki M., Ishida A., Tani H., Tokeshi M. (2016). Advances in microfluidic paper-based analytical devices for food and water analysis. *Micromachines*.

[110] Kim J., Johnson M., Hill P., Gale B. K. (2009). Microfluidic sample preparation: cell lysis and nucleic acid purification. *Integrative Biology*.

